# Adrenal, Gonadal and Peripherally Steroid Changes in Response to Extreme Physical Stress for Characterizing Load Capacity in Athletes

**DOI:** 10.3390/metabo12020091

**Published:** 2022-01-19

**Authors:** Éva Csöndör, Gellért Karvaly, Roland Ligetvári, Krisztián Kovács, Zsolt Komka, Ákos Móra, Tímea Stromájer-Rácz, András Oláh, Miklós Tóth, Pongrác Ács

**Affiliations:** 1 Department of Laboratory Medicine, Semmelweis University, 1089 Budapest, Hungary; karvaly.gellert_balazs@med.semmelweis-univ.hu (G.K.); kovacs.krisztian1@med.semmelweis-univ.hu (K.K.); tothmik1@hotmail.com (M.T.); 2 Doctoral School of Health Sciences, University of Pécs, 7621 Pécs, Hungary; roland.ligetvari@gmail.com (R.L.); akosmora86@gmail.com (Á.M.); 3 Department of Health Sciences and Sport Medicine, University of Physical Education, 1123 Budapest, Hungary; komkazsolt@gmail.com; 4 Heart and Vascular Center, Semmelweis University, 1122 Budapest, Hungary; 5 Faculty of Health Sciences, University of Pécs, 7621 Pécs, Hungary; timea.stromajer-racz@etk.pte.hu (T.S.-R.); andras.olah@etk.pte.hu (A.O.); pongrac.acs@etk.pte.hu (P.Á.); 6 János Szentágothai Research Centre, University of Pécs, 7624 Pécs, Hungary

**Keywords:** steroid, physical exercise, load capacity

## Abstract

Athletes are often exposed to extreme physical stress during training or competitions. The consequent activation of the hypothalamus–hypophysis–adrenal (HPA) axis results in intensified steroid hormone production in the adrenal cortex. We determined the impact of an acute extreme physical stress on adrenal and gonadal steroidogenesis in healthy male professional athletes (*n* = 40). The subjects underwent an extreme physical load test until total voluntary fatigue between 14:00 and 18:00 when the hormone levels are relatively stable. Blood was taken before the start (baseline), at the peak load (peak), and 30 min following completion of the exercise (recovery). The vital parameters, lactate levels, and blood levels of the 14 steroid hormones were recorded. The multivariate statistical analysis of the results revealed that all monitored hormone levels increased upon stress. Significant changes in steroid concentrations were detected at peak versus baseline, peak versus recovery, and at baseline versus recovery. The mineralocorticoid (including aldosterone and corticosterone), glucocorticoid (11-deoxycortisol and cortisol), and androgen (androstenedione, dehydroepiandrosterone, and dehydroepiandrosterone sulfate) pathways, as well as gonadal testosterone synthesis are activated simultaneously under extreme physical load. The profiling of adrenal and gonadal steroid biosynthesis in athletes may help the characterization of their loading capacity.

## 1. Introduction

The intensive mental and physical stress encountered by professional athletes trigger adaptive mechanisms, including the activation of the hypothalamus–hypophysis-–adrenal (HPA) axis [1]. Consequently, there is a risk of developing overtraining syndrome (OTS), which can lead to secondary adrenal insufficiencies [2,3]. The alterations in the circulating concentrations of endogenous (adrenocortical and gonadal) steroids have been related to individual physical capacity and condition, rendering these substances candidate biomarkers of stress tolerance and of the response to training. However, the association between adrenocortical steroidogenesis and the intensity and type of exercise or physical stress is not well understood [4,5,6].

The profiling of endogenous steroids can serve further purposes. Professional athletes must have an Athlete Biological Passport (ABP), which documents the measured testosterone (TEST), epitestosterone (EPIT), androsterone (ANDR), etiocholanolone (ETIO), 5α-androstane-3α,17β-diol (5αAdiol), and 5β-androstane-3α,17β-diol (5βAdiol) concentrations, and TEST/EPIT, ANDR/TEST, ANDR/ETIO, 5αAdiol/5βAdiol, and αAdiol/EPIT ratios. The testing of athletes includes in-competition and out-of-competition investigations, with the latter having been initiated and conducted by the World Anti-Doping Agency, the International Olympic Committee, the International Paralympic Committee in connection with the Olympic or Paralympic Games, and the Athlete’s International Federation [7,8,9]. It is also important to reveal the occurrence of latent endocrine disorders as early as possible. Specifically, increased physical activity has been associated with a positive effect on illness-related burdens, such as high blood pressure, type-II diabetes, or stroke [10]. Aldosterone (ALDO) is the primary marker of adrenal insufficiency and aldosteronism, but its concentrations can be elevated in diabetes [11,12]. Low dehydroepiandrosterone (DHEA), dehydroepiandrosterone-sulfate (DHES), dihydrotestosterone (DHTT), and TEST levels have been associated with cardiovascular disease [13,14,15]. 17α-hydroxypregnenolone (OHPE) and 17α-hydroxyprogesterone (OHPG) tests have been valuable in diagnosing congenital adrenal hyperplasia [16,17]. Metabolic syndromes, such as Addison’s or Cushing’s disease, are characterized by abnormal cortisol (CTOL) levels [18]. High androgen concentrations may be associated with hyperandrogenic syndromes [19].

Various types of physical exercise may have a different impact on the activation of adrenal and gonadal steroid synthesis. The most intensively studied substances have been CTOL and TEST, with the serum TEST/CTOL concentration ratio being widely accepted as an indicator of overloading [20,21]. Changes in CTOL and TEST levels in response to resistance exercise have also been identified [22,23]. Most guidelines on the diagnosis of OTS recommend the assessment of adrenal or gonadal steroid levels with a special emphasis on TEST, CTOL, and cortisone (CTON), as well as on TEST/CTOL ratios. Although changes in CTOL production have been described, the findings are unclear regarding how its concentration is increasing or decreasing under load [24]. Publications on the differences in the response of CTOL, DHEA, and TEST levels of trained athletes and untrained individuals have also been controversial, regarding whether there is a difference between the steroid concentrations of these two groups under load [4,24,25,26,27,28,29]. Changes in the levels of androstenedione (ADRN), CTOL, CTON, TEST, DHEA, and DHES during physical stress have been described in serum, plasma, saliva, and urine. In adolescents, the differentiation of TEST/CTOL concentration ratios resulting from exercise and puberty-related physiological factors have been described as a useful tool for the selection of young athletes, and for the optimization of their psychological and physical training program [30].

The aim of the presented study is to simultaneously elucidate the changes in the blood concentrations of 14 adrenal and gonadal steroid hormones: ALDO, ADRN, DHEA, DHES, 11-deoxycorticosterone (DCON), 11-deoxycortisol (11DC), 21-deoxycortisol (21DC), DHTT, OHPE, OHPG, corticosterone (CCON), CTOL, CTON, and TEST in professional male athletes before and during physical exercise or stress, as well as during the recovery phase.

## 2. Results

### 2.1. Evaluation of the Intensity of the Physical Load

Exercise (lactate and gas-exchange) parameters confirmed the exposure of the participants to extreme physical stress (Table 1). The lactate concentrations were significantly (*p* < 0.001) elevated at the peak of the load compared to the baseline values and were also significantly higher (*p* < 0.001) during recovery as compared to baseline.

### 2.2. Exploratory Multivariate Statistical Analysis of the Concentrations of Adrenal, Gonadal, and Peripherally Synthesized Steroids

**Figure 1:**
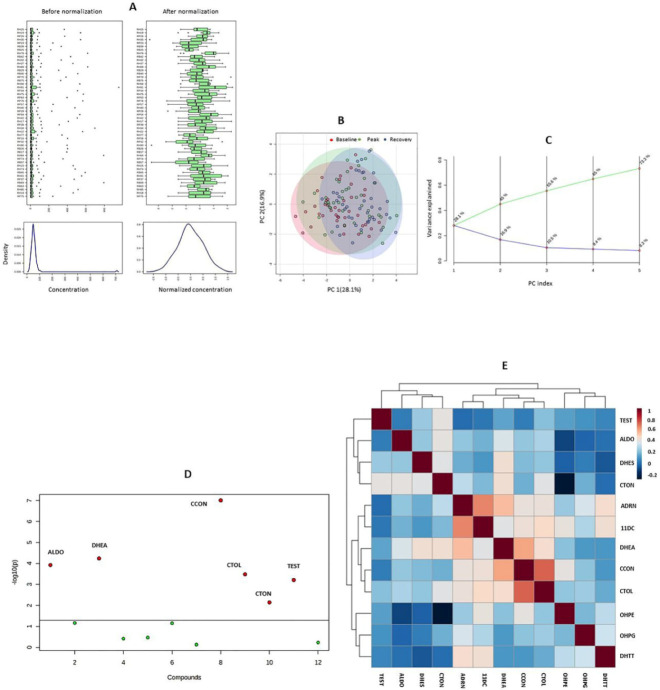
Results of the exploratory multivariate analysis. (**A**) outcomes of the glog transformation and autoscaling of endogenous steroid concentrations; (**B**) score plot of the principal component analysis (PCA); (**C**) scree plot of the PCA; (**D**) result of the nonparametric analysis of variance; (**E**) heat map showing the results of Spearman’s correlation tests. ALDO: aldosterone; ADRN: androstenedione; CCON: corticosterone; CTOL: cortisol; CTON: cortisone; 11DC: 11-deoxycortisol; DHEA: dehydroepiandrosterone; DHES: dehydroepiandrosterone-sulfate; DHTT: dihydrotestosterone; OHPE: 17α-hydroxypregnenolone; OHPG: 17α-hydroxyprogesterone; TEST: testosterone.

Principal component analysis (Figure 1) did not reveal isolated changes in the concentrations of any of the adrenal and gonadal steroids. This finding was corroborated by the results of the Kruskal–Wallis test, which indicated significant changes in the levels of the mineralocorticoids ALDO and CCON, the glucocorticoids CTOL and CTON, and the androgens DHEA and TEST (Figure 1). In terms of the categories proposed by Evans, a strong correlation was found between the concentrations of ADRN and DC11 (*r* = 0.65), as well as CCON and CTOL (*r* = 0.72) [31]. A moderate correlation was found between ADRN and DHEA (*r* = 0.52), DHEA and CCON (*r* = 0.58), CTON and DHEA (*r* = 0.41), DHES and DHEA (*r* = 0.41), DHEA and CTOL (*r* = 0.42), ADRN and DHTT (*r* = 0.42), as well as between DC11 and CTOL (r = 0.44). In all cases, the *p*-value was ≤0.01.

### 2.3. Univariate Analysis of the Changes in the Concentrations of Endogenous Steroids

In most cases, the steroid concentrations increased significantly when the exercise proceeded to the next phase, i.e., from baseline to peak and from peak to recovery (Table 2). The concentrations of ALDO, ADRN, OHPG, CTON, TEST, and DHTT rose, while CTOL concentrations declined significantly from baseline to peak. ALDO, DHEA, OHPE, CCON, and CTOL concentrations increased, while DHES, OHPG, CTON, and TEST concentrations decreased from the peak of load to recovery. During the recovery phase, higher concentrations were observed, in comparison to the baseline levels, for all of the detected steroids except for OHPG.

TEST/CTOL increased significantly from baseline to peak and decreased significantly from peak to recovery. DHEA/CTOL levels increased consistently from baseline to peak, and from peak to recovery. CCON/CTOL and CCON/DHEA were significantly higher during the recovery phase as compared to the peak of the load.

## 3. Discussion

The employed profiling approach allowed for the complex characterization of the adrenocortical and gonadal steroidogenesis following the collection of blood samples in a short time frame in response to extreme physical exercise. The outcomes of the multivariate statistical analysis indicate that the mineralocorticoid, glucocorticoid, and androgen pathways were activated simultaneously. No single steroid metabolite could be identified within any line of biosynthesis as being responsible for the observed changes. The circulating amounts of the measured steroids showed a significant increase in at least one phase of the exercise. The considerable changes in steroid concentrations of the subjects of our study point to a complex and significant physiological response. The changes in the circulating concentrations of adrenal and gonadal steroids did not indicate the selective activation of any of the steroid-producing organs (adrenal gland, testis or prostate). The most significant changes were detected during the recovery phase. In addition, none of the zones of the adrenal cortex (zona glomerulosa, zona fasciculata, or the zona reticularis) exerted signs of activation selectively.

Our findings are in accordance with those previously presented in other works performed under different settings. Smith et al. observed the elevation of DHTT concentrations in response to exercise in healthy young men [25]. Tremblay et al. assayed DHES, CTOL, and TEST before and after physical activity in trained males and found that all of the concentrations increased [32]. The enzymes 3-(or 17-)beta-hydroxysteroid dehydrogenase (EC 1.1.1.51), 11β-hydroxylase (EC1.14.15.4), 17,20-lyase (EC 1.14.14.32, CYP17A1), and 21-hydroxylase (EC 1.14.14.16, CYP21A2), are all adrenocorticotropic hormone regulated and are involved in adrenal and gonadal steroidogenesis [33,34,35,36,37]. Our results confirm that their activities increased in response to extreme physical exercise. The functions of these enzymes are also associated with various pathological conditions [38].

The ratios of TEST/CTOL and CTOL/DHEA concentrations are employed in sport physiology to evaluate the intensity of training [20,21]. The significant elevation of the CCON/CTOL ratio during recovery, compared to the baseline and peak, was primarily the result of the increased production of CCON. CTOL concentrations are less likely to display large changes due to its already high levels, the comparably low levels of its precursors, and its equilibrium with CTON. On the other hand, CCON levels could exert more pronounced changes due to its comparable concentrations with its precursors, and to the low rate of 18-hydroxycorticosterone and subsequent ALDO production [33]. Similarly, the observed increases in the CCON/DHEA concentration ratio from peak to recovery were caused by the more substantial elevation of CCON concentrations. The patterns of the observed changes in the TEST/CTOL and DHEA/CTOL concentration ratios, both of which have been established earlier as biomarkers of extreme physical stress, confirm the overload. Uchida et al. found that resistance training led to decreased TEST/CTOL ratios [39], while Heaney at al. detected significantly reduced CTOL/DHEA ratios immediately following the physical load, as well as post-exercise [40].

The results indicate that the employed methodology can be applied to the monitoring of the physiological status of professional athletes for multiple purposes. The characterization of adrenal and gonadal steroid homeostasis of young athletes can help optimize their physical load and serve as a reference for the evaluation of the impact of any future events and physiological changes on their load capacity. Potentially, steroid markers (directly measured or derived from the assayed values) may be included in biological passports, allowing for the stricter control of illicit steroid substance use. Finally, the timely detection of the occurrence of late onset endocrinological disorders can be facilitated by periodically performing a retrospective analysis of the results of steroid profiling.

Our study had limitations. The hydration status was not evaluated before and after the exercise. However, the hydrated state of the human body and exercise induced hemoconcentration can determine the hormonal response to physical activity [41,42]. Untrained (*n* = 6) male control subjects aged 18–30 who did not undergo the physical load test donated samples during the same time period to validate the findings (results not shown) and exerted stable steroid hormone levels. Due to the design and infrastructure of the study, age- and gender-matched untrained persons undergoing the same exercise protocol could not be recruited. Furthermore, a single type of physical exercise test, an extreme and acute type of load, was applied. Our study design was not suitable for the extrapolation of the obtained results to other types of load, e.g., resistance exercise, or to various types of less extreme or acute physical exercise.

## 4. Materials and Methods

### 4.1. Participants

Healthy Caucasian male adult professional handball players from Hungary were recruited. The median age of the participants was 22 years, interquartile range (IQR): 20–25, and their median body-mass index (BMI) was 23.08 kg/m^2^ (IQR: 21.87–24.46 kg/m^2^). The participants were self-reported non-smokers and did not have any known cardiovascular disease.

### 4.2. Ethics

The study was approved by the National Public Health Center of Hungary (15117–9/2018/EÜIG, 24 May 2018). All of the subjects gave their informed consent prior to beginning their participation. The study was conducted in accordance with the World Medical Association Declaration of Helsinki. M.T. was the principal investigator of this research.

### 4.3. Study Design

A non-randomized cohort study was conducted. The concentrations of adrenal, gonadal and peripherally synthesized steroids were assayed before, under and shortly after acute extreme physical stress was induced by applying extreme physical load. The loading and sampling were performed under continuous medical supervision.

### 4.4. Exercise Training

The physical stress test was executed in an exercise physiology laboratory (University of Physical Education, Department of Health Sciences and Sports Medicine, Budapest, Hungary), where the participants performed an ergospirometry (Schiller CS-200) treadmill test, reaching their maximal voluntary exhaustion, using the modified Bruce protocol (2-min warm-up at 8 km/h speed, then increased to 10 km/h and kept constant, and an elevation of 0% kept for 3 min and subsequently increased by 1.5%/min). The participants underwent the exercise between 14:00 and 18:00. The load test was performed under standard laboratory conditions. The median temperature was 24.7 °C (IQR: 22.7–25.4 °C) and the relative humidity 39.5 % (IQR: 35–42.8%). With this protocol, we wanted to standardize the extreme physical load in a laboratory environment.

### 4.5. Blood Sampling

Standard phlebotomy was performed before the load (baseline), at the maximum stress situation, as soon as possible after the load (peak), and 30 min after aborting the load (recovery) by qualified personnel. The 10 mL blood samples were taken and then centrifuged (4 °C, 1600× *g*, 15 min), and the supernatant sera were collected, frozen as soon as possible in liquid nitrogen, and stored at −80 °C until the measurement was performed.

### 4.6. Analysis

The steroid concentrations were measured at the Department of Laboratory Medicine, Semmelweis University, Budapest, Hungary, using a validated in-house liquid chromatography-tandem mass spectrometry (LC-MS/MS) method [43]. A Shimadzu Nexera X2 ultra-high performance liquid chromatograph was coupled to a Shimadzu LCMS-8060 triple quadrupole mass spectrometer. The instrument control and data acquisition were attained using the Shimadzu LabSolutions MS 5.89 software (Simkon Kft., Budapest, Hungary). For the separation, a combination of Phenomenex Kinetex XB-C18 and biphenyl (dimensions 50 × 2.1 mm for both stationary phases, particle size 1.7 µm) analytical columns, thermostated at 40 °C were used. The mobile phase consisted of water—0.1% formic acid (A) and methanol—0.1 % formic acid (B). The following gradient program was used for separation (% B): initial, 60%, 1.0 min, 60%, 4.0 min, 100%, 5.51 min, 60%. The run time was 5.5 min, and the injection volume was 5 µL.

A PowerCube gas analyzer unit, supplied by Ganshorn (Niederlauer, Germany), was used to measure the gas exchange parameters. An Omron MX2, Cardiosys Human ECG (Experimetria Kft., Budapest, Hungary) was employed for monitoring blood pressure (BP) and heart rate (HR). The HR was recorded continuously and the lactate concentrations at three time points, while the gas exchange parameters were registered continuously when under physical stress. The lactate measurements were performed on a Biosen C-line Glucose and Lactate Analyzer (Frank Diagnosztika Kft., Budapest, Hungary).

### 4.7. Data Evaluation

The respiratory quotient (RQ) was calculated as the ratio of the volume of carbon dioxide (VCO_2_) and the volume of oxygen consumed (VO_2_) per unit time (RQ = VCO_2_/VO_2_). The relative maximal VO_2_ (rel. VO_2_ max.) corresponded to the highest obtained value of VO_2_ normalized to the body weight (kg). The following criteria were used to confirm extreme physical load: (1) duration of the activity should be at least 8 min; (2) maximum HR ≥ 160–180 beats per minute, depending on the age of the participants; (3) RQ value ≥ 1.1 at the peak of the load; (4) when increasing the load, the oxygen consumption should reach its maximum; and (5) the lactate concentration at the maximum load should be 8 mmol/L or higher [44].

Basic calculations were performed using Microsoft 365 Excel. The multivariate statistical analysis was conducted using the MetaboAnalyst version 5.0 online platform (https://www.metaboanalyst.ca/ (accessed on 6 April 2021)). DCON and DC-21 were omitted from the statistical evaluation, because in most cases, their concentrations were below the detection limit. Further, a specific feature was removed when more than 25% of the measured concentrations were lower than the limit of detection. To approximate a normal distribution, the data were processed following glog transformation and autoscaling. The exploratory analysis was conducted using a principal component analysis (PCA), a nonparametric analysis of variance (Kruskal–Wallis test), and Spearman’s correlation test. A univariate statistical analysis was performed employing IBM SPSS Statistics version 25 (CNW System Integrator Co., Budapest, Hungary). A Mann–Whitney test was applied pairwise (i.e., baseline versus peak of load, peak versus recovery, and baseline versus recovery) to the non-transformed concentrations of steroids and of lactate, as well as to the concentration ratio *p* ≤ 0.05, which was considered the threshold of a statistically significant difference.

## 5. Conclusions

The profiling of endogenous steroids seems to be useful in the preliminary evaluation of the load capacity of athletes and adolescents preparing for their professional athletic careers. The presented methodology could provide complementary in-competition and out-of-competition information for the ABPs of professional athletes and may also be helpful in the early detection of late-onset forms of adrenocortical endocrine disorders, even in a subclinical stage. The findings may support the development of biomarkers to monitor the steroid homeostasis of athletes under regular physical stress.

## Figures and Tables

**Table 1:** Values of the physiological parameters and lactate concentrations as observed at the peak of the load (*n* = 40). Median values are shown along with the interquartile ranges (IQR) of heart rate (HR), lactate concentrations, relative maximal VO_2_ (rel.VO_2_ max), and respiratory quotient (RQ) in parentheses; bpm: number of beats per min.

Physiological Parameters	Observed Median (IQR)
HR-baseline (bpm)	68.5 (60.7–76.5)
HR-peak (bpm)	187.0 (183.7–191.2)
HR-recovery (bpm)	84.0 (74.5–90.5)
Lactate, baseline (mmol/L)	0.92 (0.67–1.2)
Lactate, peak (mmol/L)	10.9 (9.7–13.5)
Lactate, recovery (mmol/L)	4.2 (3.1–4.8)
Rel.VO_2_ max. (mL/kg/min)	51.3 (47.3–57.5)
Max. RQ	1.4 (1.3–1.45)

**Table 2:** Nonparametric univariate statistical comparison of the endogenous steroid concentrations measured at baseline, at the peak of the load, and during the recovery phase. Median values are shown along with the interquartile ranges (IQR), *p*-values, and the direction of changes in parentheses. Statistically significant changes in the values of the analytes are indicated by displaying the *p*-value in bold. ALDO: aldosterone; ADRN: androstenedione; CCON: corticosterone; CTOL: cortisol; CTON: cortisone; 11DC: 11-deoxycortisol; DHEA: dehydroepiandrosterone; DHES: dehydroepiandrosterone-sulfate; DHTT: dihydrotestosterone; OHPE: 17α-hydroxypregnenolone; OHPG: 17α-hydroxyprogesterone; TEST: testosterone; TEST/CTOL: testosterone–cortisol ratio; DHEA/CTOL: de dehydroepiandrosterone–cortisol ratio; CCON/CTOL: corticosterone-cortisol ratio; CCON/DHEA: corticosterone-dehydroepiandrosterone ratio; ↑: increase, ↓: decrease, -: no response.

	Baseline-Peak		Baseline-Recovery		Peak-Recovery		Median (IQR) (pmol/mL)		
	Response	*p*-Value	Response	*p*-Value	Response	*p*-Value	Baseline	Peak	Recovery
ALDO	↑	**<0.001**	**↑**	**<0.001**	**↑**	**0.010**	0.08 (0.08–0.10)	0.14 (0.08–0.32)	0.18 (0.08–0.40)
ADRN	↑	**0.037**	**↑**	**<0.001**	**-**	0.342	1.9 (1.1–2.7)	2.3 (1.4–3.2)	2.4 (1.7–3.4)
DHEA	-	0.114	**↑**	**<0.001**	**↑**	**<0.001**	10.3 (5.4–14.9)	11.7 (7.9–18.3)	18.7 (12.0–28.7)
DHES	-	0.114	**↑**	**<0.001**	**↓**	**<0.001**	6268 (5393–8622)	6702 (5895–9118)	6635 (5827–9348)
DC11	-	0.075	**↑**	**0.003**	**-**	0.758	0.14 (0.14–0.96)	0.32 (0.14–0.15)	0.46 (0.14–1.22)
OHPE	-	0.110	**↑**	**0.001**	**↑**	**0.023**	2.3 (1.7–4.8)	3.2 (1.9–5.2)	5.2 (1.9–8.5)
OHPG	↑	**0.051**	**-**	0.952	**↓**	**0.002**	1.9 (0.0–5.5)	2.5 (0.0–7.6)	1.8 (0.0–5.2)
CCON	-	0.304	**↑**	**<0.001**	**↑**	**<0.001**	9.5 (5.8–14.8)	10.7 (5.3–20.1)	26.9 (18.3–34.9)
CTOL	-	0.829	**↑**	**<0.001**	**↑**	**<0.001**	404 (325–484)	387 (293–483)	504 (415–567)
CTON	↑	**<0.001**	**↑**	**<0.001**	**↓**	**0.048**	82.2 (66.1–92.2)	100.2 (80.1–116.6)	84.4 (75.4–106.8)
TEST	↑	**<0.001**	**↑**	**0.004**	**↓**	**0.003**	19.8 (17.3–23.4)	5.1 (20.5–30.1)	21.9 (17.4–25.6)
DHTT	↑	**<0.001**	**↑**	**0.002**	**-**	0.077	2.3 (1.5–3.7)	2.6 (1.7–3.9)	2.6 (1.6–4.0)
TEST/CTOL	↑	**<0.001**	**↓**	**0.008**	**↓**	**<0.001**	0.05 (0.04–0.06)	0.06 (0.05–0.09)	0.04 (0.04–0.06)
DHEA/CTOL	↑	**0.032**	**↑**	**<0.001**	**↑**	**0.044**	0.03 (0.02–0.04)	0.04 (0.02–0.04)	0.04 (0.02–0.05)
CCON/CTOL	-	0.265	**↑**	**<0.001**	**↑**	**<0.001**	0.02 (0.02–0.04)	0.03 (0.02–0.04)	0.05 (0.04–0.07)
CCON/DHEA	-	0.166	**-**	0.197	**↑**	**<0.001**	0.91 (0.54–1.6)	0.90 (0.49–1.3)	1.4 (0.88–1.9)

## Data Availability

The data presented in this study are available on request from the corresponding author. Some variables are restricted to preserve the anonymity of study participants.
